# Deficient mismatch repair and *RAS* mutation in colorectal carcinoma patients: a retrospective study in Eastern China

**DOI:** 10.7717/peerj.4341

**Published:** 2018-02-05

**Authors:** Xiangyan Zhang, Wenwen Ran, Jie Wu, Hong Li, Huamin Liu, Lili Wang, Yujing Xiao, Xiaonan Wang, Yujun Li, Xiaoming Xing

**Affiliations:** 1 Department of Pathology, Affiliated Hospital of Qingdao University, Qingdao, China; 2 Department of Pathology, Qingdao University Basic Medicine College, Qingdao, China; 3 Department of Oncology, Affiliated Hospital of Qingdao University, Qingdao, China

**Keywords:** *KRAS* mutation, Deficient mismatch repair (dMMR ), *NRAS* mutation, Prognosis, Clinicopathological characteristics, Colorectal carcinoma

## Abstract

**Objectives:**

To investigate the frequency and prognostic role of deficient mismatch repair (dMMR) and *RAS* mutation in Chinese patients with colorectal carcinoma.

**Methods:**

Clinical and pathological information from 813 patients were reviewed and recorded. Expression of mismatch repair proteins was tested by immunohistochemistry. Mutation analyses for *RAS* gene were performed by real-time polymerase chain reaction. Correlations of mismatch repair status and *RAS* mutation status with clinicopathological characteristics and disease survival were determined.

**Results:**

The overall percentage of dMMR was 15.18% (121/797). The proportion of dMMR was higher in patients <50 years old (*p* < 0.001) and in the right side of the colon (*p* < 0.001). Deficient mismatch repair was also associated with mucinous production (*p* < 0.001), poor differentiation (*p* < 0.001), early tumor stage (*p* < 0.05) and bowel wall invasion (*p* < 0.05). The overall *RAS* mutation rate was 45.88%, including 42.56% (346/813) *KRAS* mutation and 3.69% (30/813) *NRAS* mutation (including three patients with mutations in both). *KRAS* mutation was significantly associated with mucinous production (*p* < 0.05), tumor stage (*p* < 0.05) and was higher in non-smokers (*p* < 0.05) and patients with a family history of colorectal carcinoma (*p* < 0.05). Overall, 44.63% (54/121) dMMR tumors harbored *KRAS* mutation, however, dMMR tumors were less likely to have *NRAS* mutation. Moreover, dMMR, *KRAS* and *NRAS* mutation were not prognostic factors for stage I–III colorectal carcinoma.

**Conclusions:**

This study confirms that the status of molecular markers involving mismatch repair status and *RAS* mutation reflects the specific clinicopathological characteristics of colorectal carcinoma.

## Introduction

Colorectal cancer (CRC) is the fourth most common cancer in China, with 331,300 new cases and 159,300 disease-related deaths in 2012 (Chen et al., 2016). The morbidity has increased steadily due to the growth of an aging population and the change of lifestyle in recent years, however, the exact mechanism and related predicted biomarkers are largely unknown.

During the past decades, microsatellite instability (MSI) and *RAS* mutation have been well studied as two prevalent genetic biomarkers involved in colorectal carcinogenesis. The mismatch repair (MMR) system, which includes the proteins MLH1, MSH2, MSH6 and PMS2, can repair incorrect base-pairing or unmatched DNA loops to maintain genomic stability. MSI is caused by a deficient mismatch repair (dMMR) system, which leads to a high rate of mutations in repeat sequences and accounts for approximately 15% of all CRCs as well as virtually all Lynch syndrome (LS) patients (Geiersbach & Samowitz, 2011; Marra & Boland, 1995; Zhang et al., 2016). Tumors with high level microsatellite instability (MSI-H) caused by germ line mutations or epigenetic silencing of MMR genes have unique clinicopathological characteristics (Cunningham et al., 2010). In early stage CRC, patients with MSI-H demonstrated favorable prognosis compared to those with low level of microsatellite instability (MSI-L) and microsatellite stability (MSS) (Ribic et al., 2003; Sinicrope et al., 2011), however, these patients did not benefit from fluoropyrimidine-based adjuvant chemotherapy (Ribic et al., 2003; Sargent et al., 2010).

The *RAS* gene family, the other significant biomarker, includes *KRAS*, *NRAS* and *HRAS*, and is located downstream in the epidermal growth factor receptor (EGFR) signal pathway. Mutations in the *RAS* gene, which are thought to occur early in the adenoma-carcinoma continuum, activate the *RAS*/MAPK pathway independently of EGFR activation, leading to poor response to EGFR inhibitors (Amado et al., 2008; Punt, Koopman & Vermeulen, 2016). Moreover, National Comprehensive Cancer Network (NCCN) clinical practice guidelines suggested that *KRAS* and *NRAS* gene mutations should be detected for metastatic CRC (mCRC) patients before treatment with Cetuximab and Panitumumab (Engstrom et al., 2009).

The status of dMMR and *RAS* mutation has been widely studied in western countries. The frequency of dMMR CRCs ranged from 15–20% (Giraldez et al., 2010; Sinicrope et al., 2011; Sinicrope et al., 2012), *KRAS* mutation ranged from 20–50% (De Roock et al., 2010; Naguib et al., 2010; Palomba et al., 2016; Rosty, 2013; Sasaki et al., 2016) and *NRAS* mutation was noted in less than 5% (De Roock et al., 2010; Palomba et al., 2016; Peeters et al., 2013; Russo et al., 2014). However, studies in China showed a lower frequency of dMMR compared with that in western populations, and the clinicopathological characteristics were also inconsistent (Huang et al., 2010; Jin et al., 2008; Ye et al., 2015). Although several studies reported the frequency of *KRAS* mutation in Chinese CRC patients, the number of samples was limited in most of these studies (Shen et al., 2011; Ye et al., 2015; Yunxia et al., 2010). Moreover, information about *NRAS* mutation in Chinese CRC patients was limited. Little has been studied on the association between status of dMMR and *RAS* mutation. Therefore, in the present study, we analyzed the dMMR and *RAS* mutation status of CRC patients to evaluate possible associations between dMMR, *RAS* mutation and the clinicopathological characteristics in primary colorectal carcinoma and we also attempted to explore the prognostic roles of dMMR and *RAS* mutation.

## Materials and Methods

**Figure 1:**
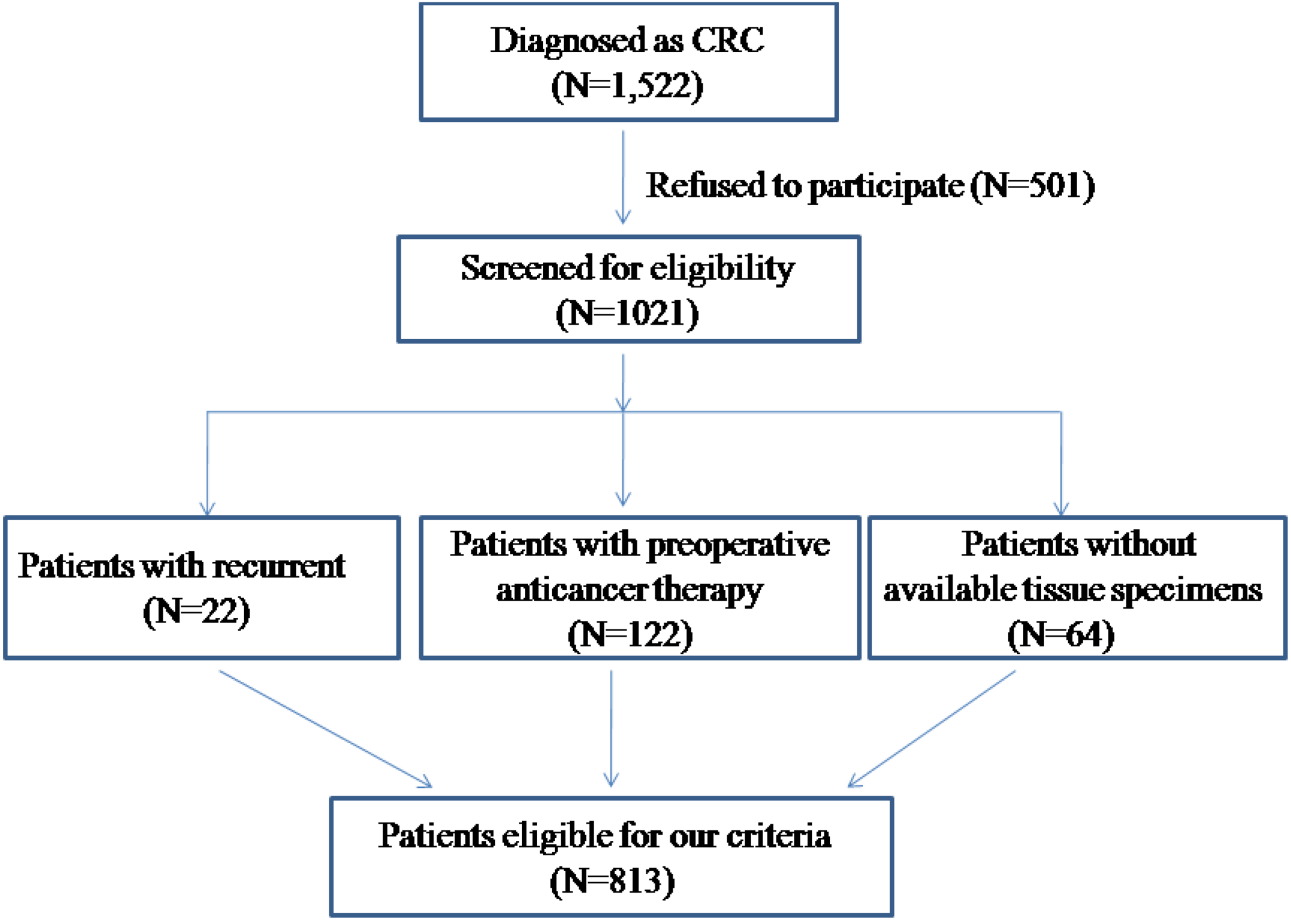
Consort diagram in patient selection.

Eight hundred and thirteen formalin-fixed, paraffin-embedded tumor specimens from CRC patients who underwent primary surgical resection from 2013 to 2016 in the Affiliated Hospital of Qingdao University were selected for this study. The patients’ selection method is presented in a consort diagram (Fig. 1). Patients who had undergone preoperative radiotherapy, chemotherapy and/or EGFR-targeted therapy were not included in this study.

The clinical and pathologic variables were extracted from medical records and pathological reports, which included age, gender, primary locations of tumor, tumor diameter, histological characteristics, TNM stage, smoking status, drinking status and family medication history. The patients were followed up until October 2017, and the data concerning cancer recurrence and patient survival were collected. Patients diagnosed with stage I–III colorectal carcinoma were used to explore the prognostic role of dMMR and *RAS* mutation with disease-free survival (DFS) and overall survival (OS).

Primary locations of tumors were divided into the right side colon (from the cecum through the transverse colon), the left side colon (from the splenic flexure through the rectosigmoid flexure) and the rectum. Tumors were staged according to the criteria of the seventh edition of the American Joint Commission on Cancer (AJCC) TNM staging system. Mucinous adenocarcinoma and signet-ring cell carcinomas were recorded as mucin-producing tumors.

The study was approved by the Ethics Committee of the Affiliated Hospital of Qingdao University (No.20130049) and all patients had signed informed consent.

### Immunohistochemistry for MMR proteins

As previously described (Lin et al., 2014b), all specimens were fixed in 10% neutral buffered formalin and embedded in paraffin blocks. 3 µm-thick tissue sections were used for immunohistochemical analysis. Immunohistochemical staining was performed on an Automated Staining System (BenchMark XT, Ventana Medical Systems, Inc., Tucson, AZ, USA) according to the manufacturer’s instructions. The ready-to-use antibodies were used as follows: MLH1 (No.M1, Ventana Medical Systems Inc, Tucson, AZ, USA, working solution), PMS2 (No.EPR3947, Ventana Medical Systems Inc, Arizona, USA, working solution), MSH2 (No.G219-1129, Ventana Medical Systems Inc, Tucson, AZ, USA, working solution), MSH6 (No.44, Ventana Medical Systems Inc, Tucson, AZ, USA, working solution).

**Figure 2:**
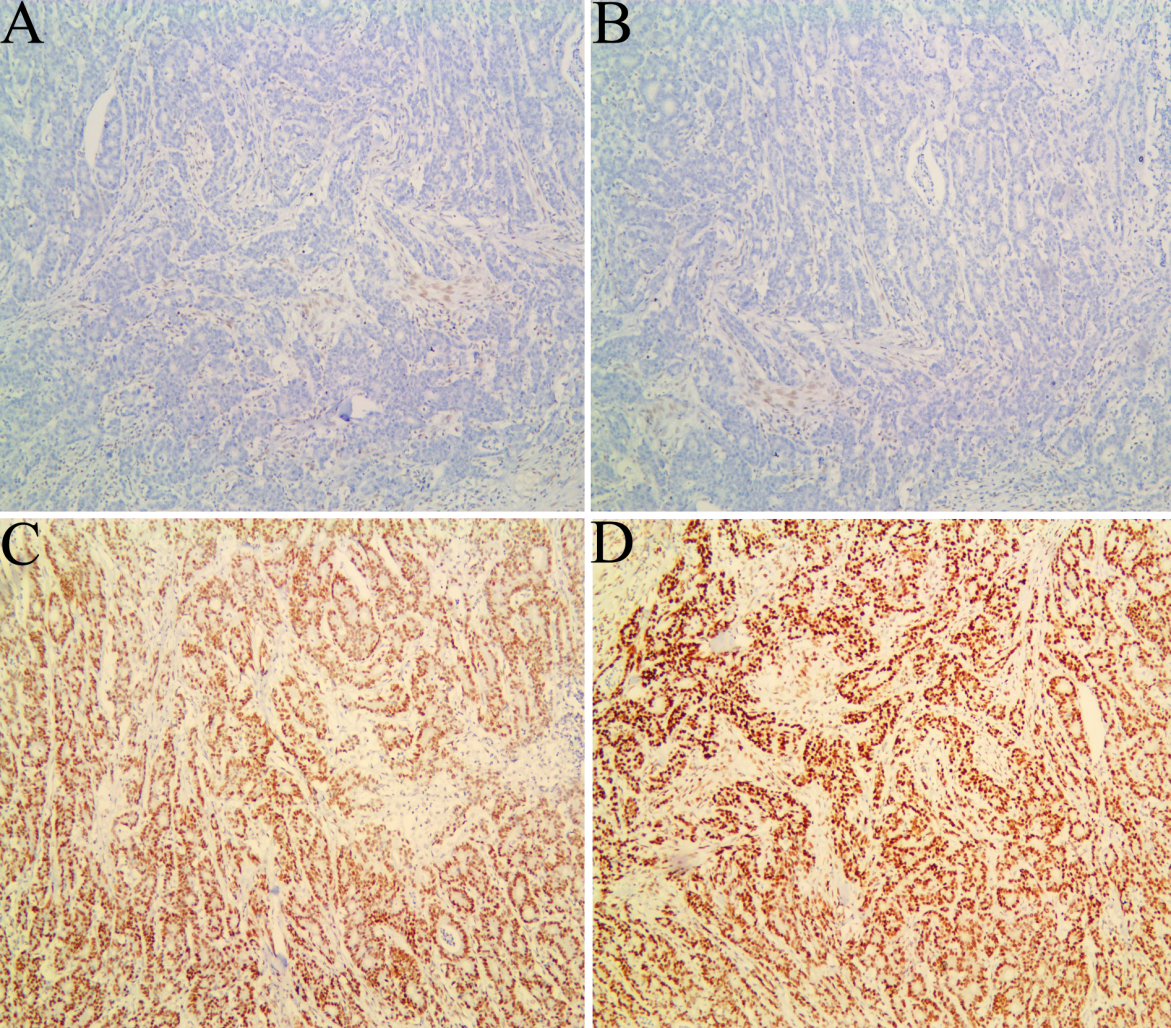
Immunohistochemical staining for mismatch repair proteins in one case of colorectal carcinoma. Tumor cells with absent MLH1 (A) and PMS2 (B) expression, and with MSH2 (C) and MSH6 (D) expression, which were regarded as deficient MMR. Note the presence of positive staining in stromal cells and lymphocyte serving as internal positive controls.

The results were analyzed by two pathologists. Any tumor cell with nuclear staining was recorded as positive staining. Intact expression for all these proteins was regarded as proficient MMR (pMMR). Protein expression was defined as abnormal when nuclear staining of tumor cells was absent in the presence of positive staining in stromal cells and lymphocytes (Fig. 2). The standard criteria for diagnosis of dMMR was as follows: dMMR in MLH1: loss of MLH1 and PMS2; dMMR in MSH2: loss of MSH2 and MSH6; dMMR in MSH6: loss of MSH6; dMMR in PMS2: loss of PMS2 (Richman, 2015).

### Analysis of *KRAS* and *NRAS* gene mutations by ARMS-PCR

Formalin-fixed, paraffin-embedded tumor sections were deparaffinized and air dried, and DNA was extracted using the Tiangen Blood and Tissue Kit (TiangenInc, Beijing, China). *KRAS* (codons12 and 13) and *NRAS* (codons12, 13 and 61) mutations were detected by amplification refractory mutation system in multiple quantitative polymerase chain reaction (ARMS-multi-qPCR) analysis with the Human *KRAS* and *NRAS* Mutation Detection kit (YuanQi Bio-Pharmaceutical Co., Ltd. Shanghai, China). The mutation points detected by this kit are listed in Supplemental Information 2. Codons of *RAS* were amplified as described previously (Dong et al., 2016). Briefly, 3 µl sample DNA was amplified in a 25 µl reaction containing 9 µl of Mix1 and 13 µl of PCRMix3. Positive and negative controls for each sample were run simultaneously. The program for the PCR amplification flanking *KRAS* mutation site was as follows: 1 cycle at 42 °C for 5 min; 1 cycle at 94 °C for 3 min; 40 cycles at (94 °C for 15 s; 60 °C for 60 s). Fluorescence signals were collected at 60 °C. The program for the PCR amplification flanking *NRAS* mutation site was as follows: 1 cycle at 42 °C for 5 min; 1 cycle at 94 °C for 3 min; 40 cycles at (94 °C for 45 s; 60 °C for 80 s). Fluorescence signals were collected at 60 °C. The mutations were identified with a specific probe labeled with Hydroxy fluorescein (FAM). Amplicons were detected using ABI7500 Fast Real-Time PCR System (Thermo Fisher Scientific Inc., Waltham, MA, US).

### Statistical analysis

Results were analyzed with SPSS 19.0 (SPSS, Inc, Chicago, IL, USA). For comparison of the frequencies among groups, the Chi-square test and the Fisher exact test were used. Survival curves for DFS and OS were estimated using Kaplan–Meier analysis with the log-rank test. Probability (*p*) value <0.05 was considered as statistical significance.

## Results

### Patient characteristics

The main characteristics of the patients are summarized in the Table 1. There were 506 (62.24%) males and 307 (37.76%) females with a mean age of 64 years. The majority of the patients (87.7%) were older than 50 years. 11.69%, 40.84%, 37.15% and 10.33% of patients presented with stage I, stage II, stage III and stage IV disease, respectively. The primary location was more common in rectum (54.49%). There were 283 (34.81%) patients with a smoking history and 165 (20.3%) patients with an alcohol in-taking history, respectively. There were 133 (16.36%) patients with mucin-productive carcinoma.

**Table 1:** Clinicopathological information of the studied patients (*n* = 813).

Characteristics	Number	(%)
Gender		
Male	506	62.24
Female	307	37.76
Age		
<50	100	12.3
≥50	713	87.7
Location		
Right side colon	181	22.26
Left side colon	189	23.25
Rectum	443	54.49
Mucin production		
With	133	16.36
Without	680	83.64
Tumor differentiation		
Poor	138	16.97
moderate	599	73.68
Well	33	4.06
Unknown	43	5.29
Tumor stage		
I	95	11.69
II	332	40.84
III	302	37.15
IV	84	10.33
Bowel wall invasion (T)		
T1	21	2.58
T2	104	12.79
T3	336	41.33
T4	352	43.3
Lymph node metastasis (N)		
N0	458	56.33
N1	203	24.97
N2	152	18.7
Distant metastasis (M)		
M0	729	89.67
M1	84	10.33
Lymphovascular invasion		
Yes	339	41.7
No	462	56.83
Unknown	12	1.47
Alcohol intake		
Ever	165	20.3
Never	648	79.7
Smoking		
Ever	283	34.81
Never	530	65.19
Colorectal family history		
Yes	48	5.9
No	337	41.45
Unknown	428	52.65

### MMR status and associations with clinicopathological characteristics

MMR status was successfully evaluated in 797 patients. 121 (15.18%) patients exhibited dMMR. The rates of dMMR deficiency in MLH1, PMS2, MSH2 and MSH6 were 9.78% (78/797), 1.25% (10/797), 3.26% (26/797) and 0.87% (7/797), respectively. The rates of deficiency in MLH1/PMS2 and MSH2/MSH6 were 11.92% (88/797) and 4.14% (33/797), respectively. The association of clinicopathological characteristics with MMR status is presented in Table 2. The proportion of dMMR was higher in patients <50 years old (*p* < 0.001). A higher rate of dMMR was found in stage II cancers (19.02%, *p* = 0.019). dMMR status was also associated with mucinous production (*p* < 0.001), poor differentiation (*p* < 0.001) and localization of the tumor to the right side of the colon (*p* < 0.001). dMMR patients had a higher propensity to bowel wall invasion (*p* = 0.018).

**Table 2:** Correlations between mismatch repair protein deficiency and clinicopathological characteristics (*n* = 797).

Characteristics	Number	dMMR		MLH1/ PMS2		MSH2/MSH6	
		Defective (%)	*P* value	Defective (%)	*P* value	Defective (%)	*P* value
Gender							
Male	495	73 (14.75)	0.662	52 (10.51)	0.561	21 (4.24)	0.853
Female	302	48 (15.89)		36 (11.92)		12 (3.97)	
Age							
<50	99	29 (29.29)	<0.001	23 (23.23)	<0.001	6 (6.06)	0.284^*^
≥50	698	92 (13.18)		65 (9.31)		27 (3.87)	
Location							
Right side colon	173	61(35.26)	<0.001	43 (24.86)	<0.001	18 (10.4)	<0.001
Left side colon	185	25 (13.51)		18 (9.73)		7 (3.78)	
Rectum	439	35 (7.97)		27 (6.15)		8 (1.82)	
Mucin production							
With	131	36 (27.48)	<0.001	25 (19.08)	<0.001	11 (8.4)	0.007
Without	666	85 (12.76)		63 (9.46)		22 (3.3)	
Tumor differentiation							
Poor	134	36 (26.87)	<0.001	24 (17.91)	<0.001	12 (8.96)	0.012^*^
Moderate	589	71 (12.05)		51(8.66)		20 (3.39)	
Well	31	4 (12.9)		3 (9.68)		1 (3.23)	
Unknown	43						
Tumor stage							
I	94	6 (6.38)	0.019	5 (5.32)	0.110	1 (1.06)	0.288^*^
II	326	62 (19.02)		45 (13.81)		17 (5.21)	
III	301	41 (13.62)		30 (9.97)		11 (3.65)	
IV	76	12 (15.79)		8 (10.52)		4 (5.26)	
Bowel wall invasion (T)							
T1	20	3 (15)	0.018	2 (10)	0.139	1 (5)	0.067^*^
T2	102	5 (4.9)		5 (4.9)		0 (0)	
T3	334	59 (17.66)		44 (13.17)		15 (4.49)	
T4	341	54 (15.83)		37 (10.85)		17 (4.98)	
Lymph node metastasis (N)							
N0	445	74 (16.63)	0.192	54 (12.13)	0.354	20 (4.49)	0.583
N1	200	31 (15.5)		22 (11)		9 (4.5)	
N2	152	16 (10.53)		12 (7.89)		4 (2.63)	
Distant metastasis (M)							
M0	721	110 (15.26)	0.550	80 (12.13)	0.88	30 (4.16)	0.929^*^
M1	76	11 (14.47)		8 (10.53)		3 (3.95)	
Lymphovascular invasion							
Yes	335	47 (14.03)	0.451	35 (10.45)	0.679	12 (3.58)	0.481
No	457	73 (15.97)		52 (11.38)		21 (4.59)	
Unknown	5						
Alcohol intake							
Ever	162	19 (11.72)	0.170	13 (8.02)	0.170	6 (3.7)	0.755
Never	635	102 (16.06)		75 (11.81)		27 (4.25)	
Smoking							
Ever	263	35 (13.31)	0.170	24 (9.13)	0.226	11 (4.18)	0.967
Never	534	86 (16.1)		64 (11.98)		22 (4.12)	
Colorectal family history							
Yes	48	11 (22.92)	0.071	5 (10.42)	0.795	6 (12.5)	0.016^*^
No	335	44 (13.13)		32 (9.55)		12 (3.58)	
Unknown	414						

**Notes.**

* Fisher’s exact test was used.

Although dMMR tumors were present more often in patients with CRC family history, no significant difference (22.92% vs 13.13%, *p* > 0.05) was found in this study. The loss of MSH2/MSH6 expression was more often observed in patients with CRC family history (12.5% vs 3.58%, *p* = 0.016). In other respects, the patients with tumors exhibiting dMMR were similar to those exhibiting pMMR.

### *RAS* gene mutation and associations with clinicopathological characteristics

*RAS* status was tested from 813 patients. The mutation rates of *KRAS* and *NRAS* were 42.56% (346/813) and 3.69% (30/813), respectively. There were three patients demonstrating mutation in both *KRAS* and *NRAS*. Patients suffering from tumors with mucinous production had a higher incidence of *KRAS* mutation compared with those having tumors without mucinous production (54.89% vs 40.18%, *p* = 0.002). A higher rate of *KRAS* mutation was found in stage II (48.49%) compared with that in stage I, stage III and stage IV (36.84%, 40.45%, 34.52%, respectively) cancers (*p* = 0.023) and in non-smokers compared with smokers (46.6% vs34.98%, *p* = 0.001). Patients with CRC family history also showed higher rate of *KRAS* mutation (54.17% vs 37.39%, *p* = 0.013). Tumors with *RAS* mutation showed lower propensity to lymph node metastasis (*p* = 0.006) and distant metastasis (*p* = 0.048). No significant associations between *KRAS* mutation and other clinicopathological characteristics were found in the present study. Meanwhile, *NRAS* mutation was not significantly associated with any clinicopathological characteristics (Table 3).

### Correlations between *RAS* mutation and MMR status

*RAS* mutation rate was slightly higher in pMMR tumors than in dMMR tumors, but failed to reach a significant difference (46.3% vs 44.63%, *p* > 0.05). There was also no obvious correlation between MMR status and *KRAS* mutation (42.3% vs 44.63%, *p* > 0.05). No *NRAS* mutation was detected in dMMR tumors. Compared with dMMR tumors, pMMR tumors had a higher propensity to harbor *NRAS* mutation (*p* = 0.009, Table 4). The distribution of MMR and *KRAS* status is shown in Supplemental Information 3. Correlation between *KRAS* gene mutation and clinicopathological characteristics in dMMR tumors is summarized in Table 5. No significant association between *KRAS* mutation and any clinicopathological characteristics were found in dMMR tumors.

### Prognostic value of dMMR and *RAS* mutation in stage I–III CRC

**Figure 3:**
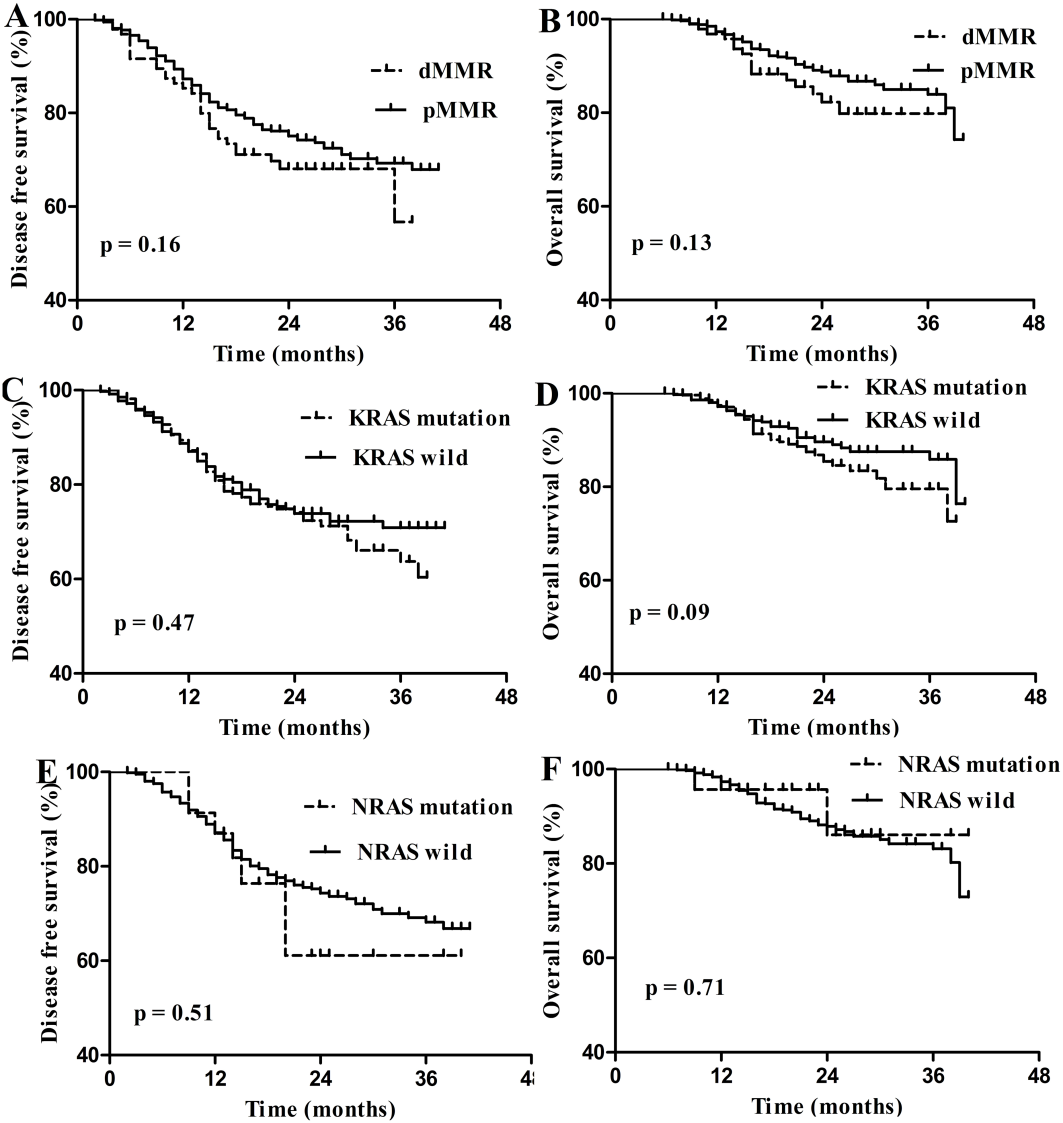
Survival curves for disease free survival (DFS) and overall survival (OS) in stage I–III colorectal carcinoma according to dMMR or RAS status. (A) Disease free survival (DFS) according to dMMR status; (B) overall survival (OS) according to dMMR status; (C) DFS according to KRAS status; (D) OS according to KRAS status; (E) DFS according to NRAS status; (F) OS according to NRAS status.

**Figure 4:**
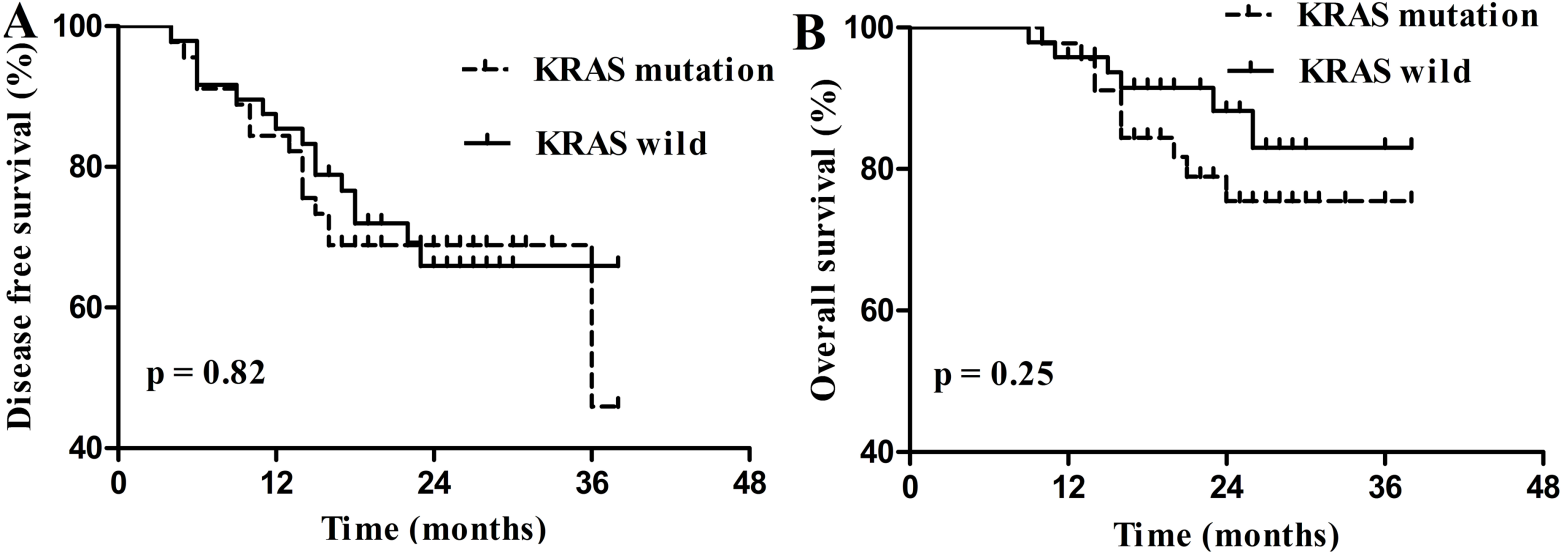
Survival curves for disease free survival (DFS) and overall survival (OS) in stage I–III dMMR colorectal carcinoma according to KRAS status. (A) Disease free survival (DFS) according to KRAS status; (B) overall survival (OS) according to KRAS status.

Of the 813 followed-up patients, 729 patients were diagnosed with stage I–III CRC, including 95 stage I patients, 332 stage II patients and 302 stage III patients. dMMR and *RAS* mutation were not prognostic for DFS and OS in stage I–III CRC (Fig. 3). Of the 121 dMMR patients, 109 patients were diagnosed with stage I–III CRC and 45.87% (50/109) patients harbored *KRAS* mutation. However, *KRAS* mutation was not prognostic factor for these patients (Fig. 4).

**Table 3:** Correlations between *RAS* gene mutations and clinicopathological characteristics (*n* = 813).

Characteristics	Number	*RAS*		*KRAS*		*NRAS*	
		Mutation (%)	*P* value	Mutation (%)	*P* value	Mutation (%)	*P* value
Gender							
Male	506	221 (43.68)	0.105	204 (40.32)	0.097	19 (3.75)	0.9
Female	307	152 (49.51)		142 (46.25)		11 (3.58)	
Age							
<50	100	38 (38)	0.091	37 (37)	0.23	1 (1)	0.161^*^
≥50	713	335 (46.98)		309 (43.34)		29 (4.07)	
Location							
Right side colon	181	91 (50.28)	0.178	88 (48.62)	0.097	3 (1.66)	0.164
Left side colon	189	77 (40.74)		71 (37.57)		6 (3.17)	
Rectum	443	205 (46.28)		187 (42.21)		21 (4.74)	
Mucin production							
With	133	74 (55.64)	0.014	73 (54.89)	0.002	1 (0.75)	0.087
Without	680	299 (43.97)		273 (40.18)		29 (4.22)	
Tumor differentiation							
Poor	138	55 (39.86)	0.315	54 (39.13)	0.604	1 (0.72)	0.093
Moderate	599	276 (46.08)		251 (41.9)		28 (4.67)	
Well	33	17 (51.52)		16 (48.48)		1 (3.03)	
Unknown	43						
Tumor stage							
I	95	41 (43.16)	0.031	35 (36.84)	0.023	6 (6.32)	0.18^*^
II	332	170 (51.2)		161 (48.49)		9 (2.71)	
III	302	133 (44.04)		122 (40.4)		14 (4.64)	
IV	84	29 (34.52)		28 (34.52)		1 (1.19)	
Bowel wall invasion (T)							
T1	21	9 (42.86)	0.36	8 (38.1)	0.158	1 (4.76)	0.36^*^
T2	104	40 (38.46)		34 (32.69)		6 (5.77)	
T3	336	154 (45.83)		146 (43.45)		9 (2.68)	
T4	352	170 (48.3)		158 (44.89)		14 (3.98)	
Lymph node metastasis (N)							
N0	458	224 (48.91)	0.006	209 (45.63)	0.079	15 (3.28)	0.265
N1	203	88 (43.35)		83 (40.89)		6 (2.96)	
N2	152	61 (40.13)		54 (35.53)		9 (5.92)	
Distant metastasis (M)							
M0	729	343 (47.05)	0.048	317 (43.48)	0.116	29 (3.98)	0.353^*^
M1	84	30 (35.71)		29 (34.52)		1 (1.19)	
Lymphovascular invasion							
Yes	339	157 (46.31)	0.763	145 (42.77)	0.825	14 (4.13)	0.623
No	462	209 (45.24)		194 (41.99)		16 (3.46)	
Unknown	12						
Alcohol intake							
Ever	165	67 (40.61)	0.128	63 (38.18)	0.203	5 (3.03)	0.615
Never	648	306 (47.22)		283 (43.67)		25 (3.86)	
Smoking							
Ever	283	109 (38.52)	0.002	99 (34.98)	0.001	10 (3.53)	0.863
Never	530	264 (49.81)		247 (46.6)		20 (3.77)	
Colorectal family history							
Yes	48	28 (58.33)	0.017	26 (54.17)	0.013	3 (6.25)	0.178^*^
No	337	135 (40.95)		126 (37.39)		9 (2.67)	
Unknown	428						

**Notes.**

* Fisher’s exact test was used.

**Table 4:** Correlations between mismatch repair protein deficiency and RAS status (*n* = 797).

MMR status	*RAS*		*KRAS*		*NRAS*	
	Mutant/tested cases (%)	*P* value	Mutant/tested cases (%)	*P* value	Mutant/tested cases (%)	*P* value
dMMR	54/121 (44.63)	0.734	54/121 (44.63)	0.635	0/121 (0)	0.009^*^
MHL1/PMS2 deficiency	39/88 (44.32)	0.725	39/88 (44.32)	0.875	0/88 (0)	0.044^*^
MSH2/MSH6 deficiency	15/33 (45.45)	0.999	15/33 (45.45)	0.72	0/33 (0)	0.391^*^
pMMR	313/676 (46.3)		286/676 (42.3)		30/676 (4.43)	

**Notes.**

* Fisher’s exact test was used.

**Table 5:** Correlations between *KRAS* gene mutations and clinicopathological characteristics in dMMR tumors (*n* = 121).

Characteristics	Number	*KRAS*	*P* value
		Mutation (%)	
Gender			
Male	73	31 (42.47)	0.555
Female	48	23 (47.91)	
Age			
<50	29	11 (37.93)	0.405
≥50	92	43 (46.74)	
Location			
Right side colon	61	26 (42.62)	0.891
Left side colon	25	12 (48)	
Rectum	35	16 (45.71)	
Mucin production			
With	36	20 (55.56)	0.116
Without	85	34 (40)	
Tumor differentiation			
Poor	36	10 (27.78)	0.099^*^
Moderate	71	35 (49.3)	
Well	4	2 (50)	
Unknown	10		
Tumor stage			
I	6	2 (33.33)	0.277^*^
II	62	33 (53.2)	
III	41	15 (36.59)	
IV	12	4 (33.33)	
Bowel wall invasion (T)			
T1	3	2 (66.67)	0.179^*^
T2	5	0 (0)	
T3	59	26 (44.07)	
T4	54	26 (48.15)	
Lymph node metastasis (N)			
N0	74	38 (51.35)	0.056
N1	31	13 (41.94)	
N2	16	3 (18.75)	
Distant metastasis (M)			
M0	110	50 (45.45)	0.753^*^
M1	11	4 (36.36)	
Lymphovascular invasion			
Yes	47	21 (44.68)	0.927
No	73	32 (43.83)	
Unknown	1		
Alcohol intake			
Ever	19	9 (47.37)	0.855
Never	102	46 (45.1)	
Smoking			
Ever	35	14 (40)	0.514
Never	86	40 (46.51)	
Colorectal family history			
Yes	11	5 (45.45)	0.589
No	44	24 (54.55)	
Unknown	66		

**Notes.**

* Fisher’s exact test was used.

## Discussion

As prognostic and predictive biomarkers, MMR deficiency and *RAS* mutation are important for clinical treatment and prognosis of CRC patients. Compared with pMMR, patients with dMMR CRCs are reported to have unique clinicopathological characteristics such as poor differentiation, early stage, increased tumor-infiltrating lymphocytes and better clinical outcome (Brenner, Kloor & Pox, 2014; Korphaisarn et al., 2015; Ribic et al., 2003). The *RAS* gene is a predictive biomarker for the resistance to anti-EGFR monoclonal antibody (MoAb) treatment in mCRCs (Amado et al., 2008; Punt, Koopman & Vermeulen, 2016). However, geographic and racial differences between Chinese and other countries were reported (Huang et al., 2010; Ismael et al., 2017; Kim et al., 2007; Vasovcak et al., 2011; Ye et al., 2015), which need to be validated with large sample amounts. Furthermore, data regarding *RAS* mutation frequency and dMMR CRC is not consistent in China. Thus, we designed this study in the Chinese population aiming to explore the relationship between the *RAS* mutation, MMR status and clinicopathological parameters, also expecting to find some prognostic and predictive biomarkers for CRC.

Our results demonstrated an overall MMR deficiency rate of 15.18%, which is within the established range of 15–21% (Giraldez et al., 2010; Sinicrope et al., 2012; Carethers et al., 2004; Cushman-Vokoun et al., 2013), but slightly higher than that reported from other Chinese populations (Huang et al., 2010; Jin et al., 2008; Ye et al., 2015). Reports from Korea (Jung et al., 2012) and Japan (Kadowaki et al., 2015) which used PCR-based MSI testing also showed that the frequencies of MSI-H CRCs were around 10%. This discrepancy can be explained by the different detective methods to some extent. Compared with PCR-based MSI testing examination, immunohistochemistry is thought to be easily available and time-saving. Furthermore, immunohistochemistry may detect MMR-deficient cases that can be potentially missed by PCR-based MSI testing (Shia, 2008).

Correlations between dMMR status and clinicopathological characteristics were controversial (Ismael et al., 2017; Jin et al., 2008; Ribic et al., 2003; Sinicrope et al., 2011). Reports from three independent Chinese groups (Huang et al., 2010; Jin et al., 2008; Ye et al., 2015) indicated that dMMR had specific associations such as female gender, right sided colon tumors and mucious tumors. In a study including 1,063 CRCs, Lin et al. (2014a) observed that MSI was associated not only with gender, tumor location and mucin production, but also with tumor differentiation and tumor stage. In our current study, we found patients younger than 50 tended to be dMMR. These diverse findings may be attributed to different criteria for age division, ethnicities, environmental factors as well as the specificity and sensitivity of the detection methods.

In our study, there was a correlation between MSH2/MSH6 deficiency and family history of CRC, but not MLH1/PMS2 deficiency. In addition, according to the Bethesda criteria (Burt et al., 2010), 12 CRCs were diagnosed with LS. In MSH2/MSH6 deficient CRCs, 33.3% (6/18) were LS, while in MLH1/PMS2 defective cases, 13.95% (6/43) were LS, suggesting MSH2/MSH6 deficient patients had higher opportunity to be diagnosed with LS. Some of the recent studies may help to explain this finding: the majority dMMR CRCs were caused by inactivation of MLH1 and more than 70% MLH1 deficiency was caused by *MLH1* promoter hypermethylation (Hampel et al., 2005), which could distinguish sporadic dMMR CRCs from LS cases, therefore, most MLH1 defective tumors were sporadic CRC. Another interesting phenomenon in our investigation is that we found most patients’ family medical history was unclear and they did not know whether other family members had polyps removed, moreover, many cancers might be prevented by early stage colonoscopy, so the family history may be deceptive (Hampel, 2014). Therefore, screening strategy based on family history may be improper. All patients with newly diagnosed CRC should be screened for LS (Hampel, 2014). Inconsistent with previous studies, which indicated that patients with dMMR tumors had significantly better survival than that of pMMR patients (Des Guetz et al., 2009; Korphaisarn et al., 2015; Lanza et al., 2006), our study showed that dMMR was not a prognostic factor for patients with stage I–III CRC, although the incidence of dMMR in stage III disease was lower, suggesting that dMMR tumors had lower propensity to metastasize.

In the present study, the mutation rates of *KRAS* and *NRAS* are 42.56% and 3.69%, respectively. The *KRAS* mutation rate is significantly higher than the value of 20.7% among 314 CRC patients from Taiwan, China (Liou et al., 2011), 22% among 202 CRC patients from the England (Naguib et al., 2010), 30.1% among 392 CRC patients from Switzerland (Zlobec et al., 2010), but similar to that previously reported in Guangzhou, China (43.9%, 25/57) (Mao et al., 2012). Several factors may lead to such differences, such as sample size, dietary and lifestyle factors, as well as racial and/or environmental differences. Furthermore, we detected the coding sequence of codon12 and codon13 in exon 2 of the *KRAS* gene, which may help to explain the higher percentage of *KRAS* mutation than those detected in codon12 only. Except for exon 2, recent studies have shown 5–10% of tumors harbored exon 3 or exon 4 mutation (Janakiraman et al., 2010; Lin et al., 2014a), which would also result in resistance to anti-EGFR inhibitors. Therefore, extending the detection spectrum of *RAS* might help to optimize the selection of the CRC patients to receive anti-EGFR MoAbs.

The frequency of *KRAS* mutation has been reported to be associated with age, gender, differentiation and tumor stage (Gao et al., 2012; Li et al., 2011; Ye et al., 2015; Yunxia et al., 2010; Zhu et al., 2012). Inconsistent with these results, our study showed that *KRAS* mutation was associated with mucin production, tumor stage, non-smoking and CRC family history. *RAS* mutated tumors showed lower propensity to lymph node and distant metastasis. No convincing evidence demonstrates that *KRAS* mutation is an independent prognostic factor for CRC (Jin et al., 2008; Palomba et al., 2016; Russo et al., 2014; Yunxia et al., 2010). In the present study, no associations of *KRAS* mutation with DFS and OS were found in patients with stage I–III CRC. Further studies based on longer follow-up time and larger sample size are needed to confirm this conclusion.

In our study, the percentage of the four tumor subgroups, including dMMR/*KRAS* mutation, dMMR/*KRAS* wild-type, pMMR/*KRAS* mutation and pMMR/*KRAS* wild-type tumors was 6.78%, 8.4%, 35.88%, 48.94%, respectively, which is similar to the data reported by a study from Beijing, China (Ye et al., 2015). According to recent reports (Nash et al., 2009; Roth et al., 2010), patients with a MSS/*KRAS* mutant tumor had the worst survival than the other three groups. Therefore, dMMR and *KRAS* markers may provide a foundation for developing a molecular prognostic scoring system for CRC patients in the future.

Previous studies have shown that pMMR patients tended to harbor more *KRAS* mutation than dMMR patients (Naguib et al., 2010; Ye et al., 2015). One hypothesis for this result is that *BRAF* and *KRAS* mutations were almost mutually exclusive in CRC and MSI tumors are more likely to harbor a *BRAF* mutation, so MSS tumors might harbor more *KRAS* mutations (Naguib et al., 2010). However, in the present study, we did not find any differences in *KRAS* mutation between pMMR and dMMR tumors, and further studies based on larger sample size are needed to explore this controversy in Chinese CRCs.

Additionally, our study provided an opportunity to investigate the status of *KRAS* mutation in Chinese dMMR patients. *KRAS* mutation presented in 44.63% dMMR patients in our study, similar to previous studies in western countries (Cushman-Vokoun et al., 2013; Oliveira, 2004). All of these results indicate that *KRAS* mutation could be quite common in dMMR tumors. There were no associations between *KRAS* mutation and clinicopathologic characteristics in dMMR tumors. A study conducted by Nash et al. (2009) indicated that *KRAS* status was an independent prognostic factor in early stage MSI CRC patients. Moreover, MSI patients with wild-type *KRAS* and *BRAF* tumors have more favorable prognosis than patients with mutated *KRAS* or *BRAF* tumors in early stage CRC (De Cuba et al., 2016; Phipps et al., 2015). However, we did not find *KRAS* mutation as a prognostic factor for dMMR patients with stage I–III CRC.

*NRAS*, as one of the *RAS* family, showed close relations with *KRAS*. Unlike *KRAS*, *NRAS* mutation was rarely detected in CRC patients. In our study, the mutation rate of *NRAS* was 3.69%, similar to previous reports (Chang et al., 2016; Irahara et al., 2010; Palomba et al., 2016; Peeters et al., 2013). Moreover, we observed 25/388 *KRAS* wild-type tumors with *NRAS* mutation, which can partially help to explain the resistance to anti-EGFR MoAb in some *KRAS* wild-type patients. Considering the heavy financial burden in MoAb treatment in CRC patients, *NRAS* mutation should be tested before MoAb treatment in *KRAS* wild-type tumors. Another interesting phenomenon is that no *NRAS* mutation was detected in dMMR patients, which suggested *NRAS* mutation might be mutually exclusive with dMMR. Meanwhile, *NRAS* mutation was not significantly associated with any clinicopathologic characteristics in our study.

However, our results should be elucidated with consideration of its limitations: first, the sample size was relatively small, rendering some findings inconclusive; second, we used a commercially available kit authenticated by China Food and Drug Administration (CFDA) and the mutation subgroups were uncertain. A study conducted by Lin et al. (2014a) demonstrated that mutation in *KRAS* codon12 was associated with significantly poorer outcome than mutations elsewhere or wild-type *KRAS*. Therefore, the subgroup of mutation codons should be carefully explored in future; third, we did not collect data of clinical management, therefore, the influence of clinical treatment for survival was uncertain.

## Conclusion

In conclusion, this was an exploratory analysis of correlations between *RAS* mutation and MMR status with clinicopathological characteristics in Eastern Chinese CRC patients. The status of these molecular markers, involving MLH1/PMS2, MSH2/MSH6, *KRAS* and *NRAS* mutation, reflects the specific clinicopathological characteristics of CRC. More comprehensive molecular classification and survival analysis should be explored in future experiments.

##  Supplemental Information

10.7717/peerj.4341/supp-1 Supplemental Information 1 Raw data of 813 cases

10.7717/peerj.4341/supp-2 Supplemental Information 2 The mutation points that detected by our kit named the Human *KRAS* and *NRAS* Mutation Detection kit We listed the mutation points that detected by our kit named the Human *KRAS* and *NRAS* Mutation Detection kit (YuanQi Bio-Pharmaceutical Co., Ltd. Shanghai, China).

10.7717/peerj.4341/supp-3 Supplemental Information 3 The distribution of mismatch repair protein and *KRAS* mutation in colorectal cancer patients (*n* = 797) The distribution of mismatch repair protein and *KRAS* mutation in colorectal cancer patients.

## Additional Information and Declarations

### Data Availability

The following information was supplied regarding data availability: The raw data has been provided in the  Supplemental Files.
