# Supplementary material for: Deficient mismatch repair and RAS mutation in colorectal carcinoma patients: a retrospective study in Eastern China
Source: PeerJ. 2018 Feb 5;6:e4341. doi: 10.7717/peerj.4341 (PMC5804321; doi:10.7717/peerj.4341)
Supplement: Supplemental Information 2 — We listed the mutation points that detected by our kit named the Human KRAS and NRAS Mutation Detection kit (YuanQi Bio-Pharmaceutical Co., Ltd. Shanghai, China). [file peerj-06-4341-s002.docx]

We listed the mutation points that detected by our kit named the Human *KRAS* and *NRAS* Mutation Detection kit (YuanQi Bio-Pharmaceutical Co., Ltd. Shanghai, China).

| Mutation | Base change |
| --- | --- |
| KRAS-12Gly→Asp | GGT→GAT |
| KRAS-12Gly→Ala | GGT→GCT |
| KRAS-12Gly→Ser | GGT→GTT |
| KRAS-12Gly→Ser | GGT→AGT |
| KRAS-12Gly→Arg | GGT→CGT |
| KRAS-12Gly→Cys | GGT→TGT |
| KRAS-13Gly→Asp | GGC→GAC |
| KRAS-13Gly→Cys | GGC→TGC |
| NRAS-12Gly→Ser | GGT→AGT |
| NRAS-12Gly→Cys | GGT→TGT |
| NRAS-12Gly→Asp | GGT→GAT |
| NRAS-12Gly→Ala | GGT→GCT |
| NRAS-12Gly→Val | GGT→GTT |
| NRAS-13Gly→Arg | GGT→CGT |
| NRAS-13Gly→Asp | GGT→GAT |
| NRAS-13Gly→Val | GGT→GTT |
| NRAS-61Qln→Lys | CAA→AAA |
| NRAS-61Qln→Arg | CAA→CGA |
| NRAS-61Qln→Leu | CAA→CTA |
| NRAS-61Qln→His1 | CAA→CAC |
| NRAS-61Qln→His2 | CAA→CAT |
