# Supplementary figures and images for: Deficient mismatch repair and RAS mutation in colorectal carcinoma patients: a retrospective study in Eastern China
Source: PeerJ. 2018 Feb 5;6:e4341. doi: 10.7717/peerj.4341 (PMC5804321; doi:10.7717/peerj.4341)

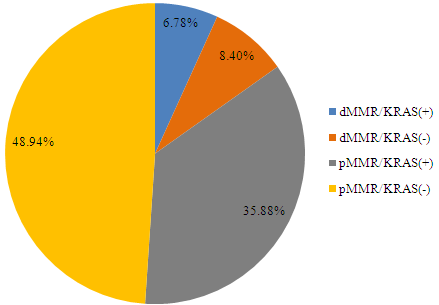

Supplement: Supplemental Information 3 — The distribution of mismatch repair protein and KRAS mutation in colorectal cancer patients. [file peerj-06-4341-s003.png]
